# How to wake a giant

**DOI:** 10.18632/oncotarget.5112

**Published:** 2015-08-06

**Authors:** Ines Liebscher, Kelly R. Monk, Torsten Schöneberg

**Affiliations:** Institute of Biochemistry, Medical Faculty, University of Leipzig, Leipzig, Germany

**Keywords:** adhesion GPCR, tethered peptide agonist, signal transduction

Research on G protein-coupled receptors (GPCRs, 7TM receptors) has attracted remarkable interest throughout the last few decades. They are involved in several human diseases and their accessibility for pharmaceutical intervention has made them the number one drug target [1]. As the de-orphanization of every receptor harbors a potential new therapeutic option, uncharacterized GPCRs are in fact a true treasure chest. While the majority of studies have been performed on Rhodopsin-like GPCRs, the whole class of Adhesion GPCRs (aGPCRs) remained orphan for a long time, even though their involvement in human syndromes and diseases including cancer has been widely demonstrated [2].

**Figure 1:**
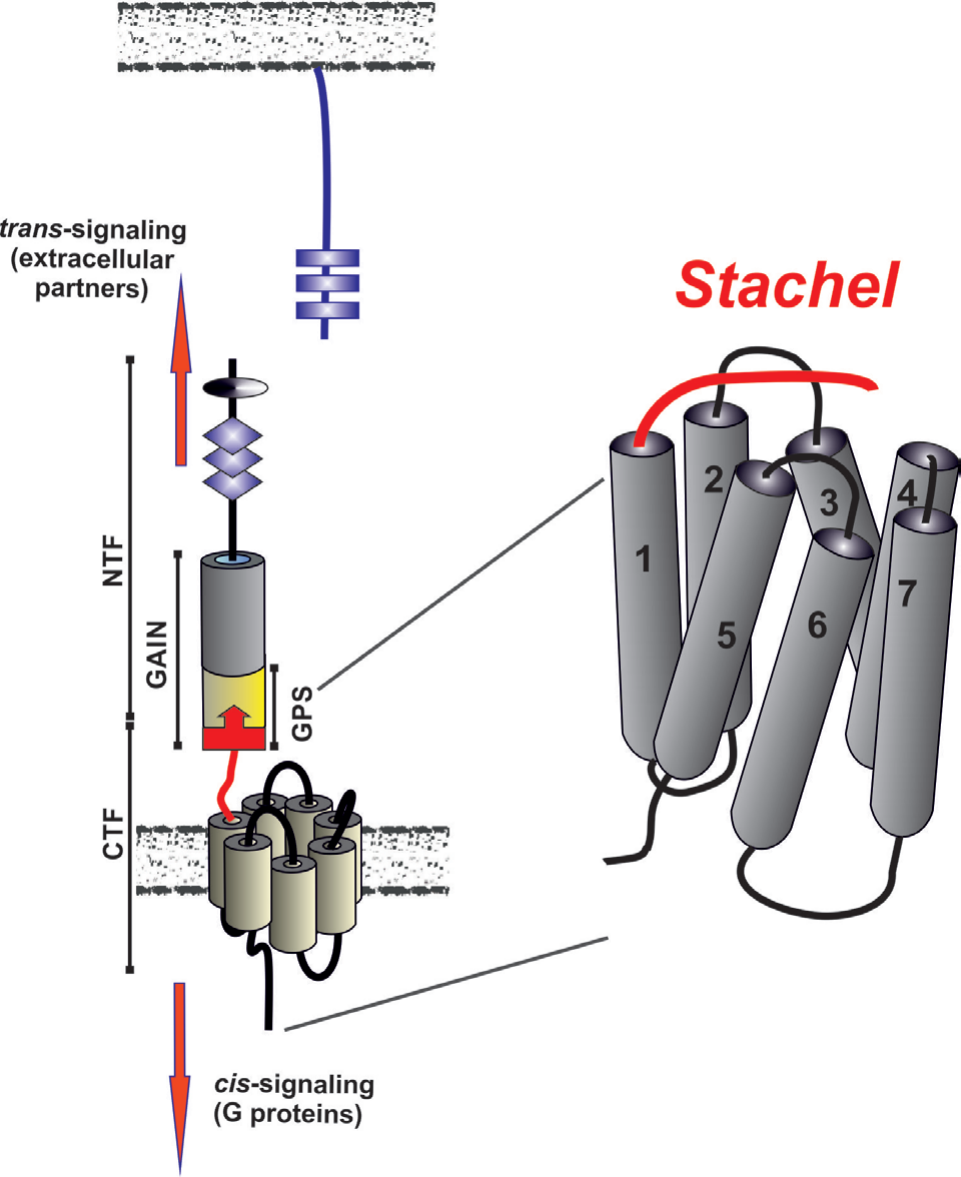
Activation mechanism of aGPCRs AGPCRs are gigantic molecules that share characteristic features as a GPCR autoproteolysis inducing (GAIN) domain which harbors the GPCR proteolytic site (GPS), at which the receptor is cleaved into an N-terminal (NTF) and a C-terminal fragment (CTF). A tethered peptide agonist called the *Stachel* sequence (red) or derived peptides activate the receptor.

With up to 6,500 amino acids, aGPCRs are giants among the 7TM receptors. The large modular N terminus in most representatives of this class undergoes an autoproteolytic cleavage event resulting in two functional parts - an N-terminal fragment (NTF) and a C-terminal fragment (CTF) (Figure 1). This cleavage event takes place at a highly conserved motif within the GPCR proteolytic site (GPS), which is part of a domain common for aGPCRs, the GPCR proteolysis-inducing (GAIN) domain. Several domains (e.g. EGF, leucine-rich domain) within the NTF are the reason that aGPCRs were initially identified as cell differentiation markers and adhesion molecules, while functions as GPCRs was mainly suggested based on sequence homology. Recently, the functional interaction between G proteins and aGPCRs was demonstrated showing that aGPCRs, as other GPCRs, can couple to different G proteins [reviewed in 2].

Knowing the intracellular signaling pathway of the giants formed the basis for examining the activation mechanism. A groundbreaking observation was that receptor mutants lacking the NTF show constitutive activity, leaving two possible explanations: 1) the NTF acts as a tethered inverse agonist or 2) the NTF blocks an encrypted tethered agonist [discussed in 3].

A recent study now provides evidences that the N termini of aGPCRs contain their own agonists and self-activate the transmembrane domain upon structural changes of the NTF. Due to the position of this agonistic region at the end of the CTF and its proclaimed piercing interaction with the 7TM, we called the tethered agonist the *Stachel* sequence - the German word for “stinger” (Figure 1) [4]. The *Stachel* region correlates with the last beta-sheet of the proposed crystal structure of the GAIN domain [5]. However, according to this structure, this last beta-sheet lies buried within other beta-sheets giving rise to the question of how the agonist is exposed. Given the cleavage motif as a natural break point, removal of the NTF seems likely, yet it remains to be proven that this actually happens *in vivo*.

There are a number of proteins that bind to the NTF, among them extracellular matrix molecules like collagen and laminin, which do not display characteristic features of an agonist. Collagens and laminins have been shown to modulate G-protein signaling of some aGPCRs. For example, one GPR126 binding partner, laminin 211, inhibits cAMP production under static conditions. However, upon application of shaking or vibration to the cell culture, an increase in cAMP accumulation was observed [6]. Analysis of a corresponding zebrafish model provided evidence that the physiological correlate of this mechanic force would be the polymerization of laminin 211. It is yet unclear whether this mechanic force will lead to the removal of the NTF as a cause of receptor activation. Interestingly, involvement in mechanosensation has also been suggested for other aGPCRs [7].

The identification of G-protein coupling as one important signaling pathway of aGPCRs (*cis*-signaling) and their activation by a tethered agonist mechanism were important steps towards understanding the physiology of this previously enigmatic receptor class. However, the NTF is most likely capable of an independent *trans*-signaling mediated by cell-cell contacts [reviewed in 3]. The recent discovery of the activation mechanism and metabotropic signal transduction of aGPCRs now allows for rational ligand design and will promote studying the physiology and therapeutic usefulness of this emerging group of GPCRs in many fields including developmental biology, immunology, neurobiology and tumorigenesis.
